# Genome Sequence of *Chlamydia suis* MD56, Isolated from the Conjunctiva of a Weaned Piglet

**DOI:** 10.1128/genomeA.00425-14

**Published:** 2014-05-08

**Authors:** Manuela Donati, Heather Huot-Creasy, Michael Humphrys, Maria Di Paolo, Antonietta Di Francesco, Garry S. A. Myers

**Affiliations:** a DIMES, Section of Microbiology, University of Bologna, Bologna, Italy; b Institute for Genome Sciences, University of Maryland School of Medicine, Baltimore, Maryland, USA; c Department of Veterinary Medical Sciences, University of Bologna, Ozzano dell’Emilia, Bologna, Italy

## Abstract

*Chlamydia suis* is a natural pathogen of pigs (*Sus scrofa*) and causes conjunctivitis, pneumonia, enteritis, and various reproductive disorders that adversely impact this economically important animal. Here, we report the first *C. suis* genome, that of *C. suis* MD56, isolated from a conjunctival swab of a weaned piglet.

## GENOME ANNOUNCEMENT

Members of the genus *Chlamydia* are obligate intracellular bacterial pathogens responsible for a variety of diseases in both humans and animals (1). *Chlamydia suis* infects pigs (*Sus scrofa*) and is associated with porcine conjunctivitis, rhinitis, pneumonia, enteritis, pericarditis, polyarthritis, polyserositis, various reproductive disorders, and inferior semen quality (2). *C. suis* infections appear to be common in both wild and domesticated pig herds and often occur in mixed infections with *Chlamydia abortus* and *Chlamydia pecorum* (2).

We sequenced *C. suis* MD56, originally isolated in 2009 from a weaned piglet (Udine, Friuli-Venezia Giulia, Italy) with conjunctivitis. A draft MD56 genome sequence was determined using Illumina sequencing chemistry on an Illumina GAII instrument, producing 3,136,872 total reads. These reads were assembled into a draft genome using Velvet (version 1.1) (3). The MD56 draft genome consists of 47 contigs, representing 160× coverage. Gene identification and annotation were performed as previously described (4). Functional assignment, identification of membrane-spanning domains, determination of paralogous gene families, and identification of regions of unusual nucleotide composition were also performed as previously described (4). The MD56 genome is 1,074,340 bp and contains 933 coding sequences (CDSs). To our knowledge, this is the first *C. suis* genome to be reported. One assembled contig exhibits high homology to the conserved 7.5-kb chlamydial plasmid.

The chlamydial plasticity zone (PZ) is a region at the replication terminus of the chromosome that encapsulates much of the observed interspecies chlamydial variation (5, 6). This heterogeneous region includes putative chlamydial virulence factors that may play a role in host tropism or niche specificity (7). The *C. suis* plasticity zone is similar to the *Chlamydia trachomatis* and *Chlamydia muridarum* PZs in both relative size and gene organization. *C. suis* possesses two copies of the chlamydial cytotoxin ortholog, whereas *C. muridarum* has three copies and *C. trachomatis* has only gene decay fragments. Both *C. suis* and *C. trachomatis* have the *trpBA* operon at the 3′ end of the PZ and lack the *guaBA* operon found in the same location in *C. muridarum*.

### Nucleotide sequence accession numbers.

This whole-genome shotgun project has been deposited in DDBJ/ENA/GenBank under the accession no. AYKJ00000000. The version described in this paper is the first version, AYKJ01000000.

## References

[1] Kuo CC Stephens R 2011 Family I. *Chlamydiaceae*, p 845 *In* Krieg NR Staley JT Brown DR Hedlund BP Paster BJ Ward NL Ludwig W Whitman WB (ed), Bergey’s manual of systematic bacteriology, vol 4, 2nd ed. Springer Verlag, New York, NY.

[2] Schautteet K Vanrompay D 2011 *Chlamydiaceae* infections in pig. Vet. Res. 42:29. 10.1186/1297-9716-42-29 21314912 PMC3041669

[3] Zerbino DR Birney E 2008 Velvet: algorithms for *de novo* short read assembly using de Bruijn graphs. Genome Res. 18:821–829. 10.1101/gr.074492.107 18349386 PMC2336801

[4] Galens K Orvis J Daugherty S Creasy HH Angiuoli S White O Wortman J Mahurkar A Giglio MG 2011 The IGS standard operating procedure for automated prokaryotic annotation. Stand. Genomic Sci 4:244–251. 10.4056/sigs.1223234 21677861 PMC3111993

[5] Read TD Brunham RC Shen C Gill SR Heidelberg JF White O Hickey EK Peterson J Utterback T Berry K Bass S Linher K Weidman J Khouri H Craven B Bowman C Dodson R Gwinn M Nelson W DeBoy R Kolonay J McClarty G Salzberg SL Eisen J Fraser CM 2000 Genome sequences of *Chlamydia trachomatis* MoPn and *Chlamydia pneumoniae* AR39. Nucleic Acids Res. 28:1397–1406. 10.1093/nar/28.6.1397 10684935 PMC111046

[6] Read TD Myers GS Brunham RC Nelson WC Paulsen IT Heidelberg J Holtzapple E Khouri H Federova NB Carty HA Umayam LA Haft DH Peterson J Beanan MJ White O Salzberg SL Hsia RC McClarty G Rank RG Bavoil PM Fraser CM 2003 Genome sequence of *Chlamydophila caviae* (*Chlamydia psittaci* GPIC): examining the role of niche-specific genes in the evolution of the *Chlamydiaceae*. Nucleic Acids Res. 31:2134–2147. 10.1093/nar/gkg321 12682364 PMC153749

[7] Myers GSA Crabtree J Huot-Creasy H 2012 Deep and wide: comparative genomics of *Chlamydia*, p 27–50 *In* Tan M Bavoil P (ed), Intracellular pathogens I: *Chlamydiales*. ASM Press, Washington, DC

